# AI‐enabled engineering of hesperidin/ursodeoxycholic acid nanomedicine for synergistic treatment of drug‐induced liver injury

**DOI:** 10.1002/smo2.70101

**Published:** 2026-09-03

**Authors:** Kai Xiao, Meng Fan, Haoyu Zheng, Boyuan Gu, Shaowen Wang, Ziping Wu, Shiming Zhang, Xin Zhao, Wensheng Zhang, Quanxin Ning, Sigen A, Chao Yang, Huayi Sun, Ren Mo, Dan Shao

**Affiliations:** ^1^ School of Medicine South China University of Technology Guangzhou Guangdong China; ^2^ School of Electronic and Computer Engineering Peking University Shenzhen Guangdong China; ^3^ Department of Urology Inner Mongolia Autonomous Region People's Hospital Hohhot Inner Mongolia China; ^4^ Institutes of Biomedical Sciences Inner Mongolia University Hohhot Inner Mongolia China; ^5^ State Key Laboratory of Oral Diseases & National Center for Stomatology & National Clinical Research Center for Oral Diseases West China Hospital of Stomatology Sichuan University Chengdu Sichuan China; ^6^ Department of General Surgery Shengjing Hospital of China Medical University Shenyang Liaoning China; ^7^ Department of Orthopedics Center for Orthopedic Surgery The Third Affiliated Hospital of Southern Medical University Guangzhou Guangdong China; ^8^ Department of Hepatobiliary‐Pancreatic‐Splenic Surgery Inner Mongolia Autonomous Region People's Hospital Hohhot Inner Mongolia China

**Keywords:** API‐target interaction, artificial intelligence, drug‐induced liver injury, nanomedicine, therapeutic combination

## Abstract

Drug‐induced liver injury (DILI) is a complex and intractable disease because existing anti‐oxidate therapies in clinic fail to modulate multiple pathological pathways concurrently. Here, we present a direction‐aware framework that integrates disease‐network analysis, AI‐guided molecular screening, and self‐assembled nanomedicine design for precise protection of DILI. Time‐resolved transcriptomic profiling of DILI identifies two complementary repair axes: the suppression of cytokine‐cytokine receptor signaling for inflammation control together with the activation of glutathione biosynthesis for antioxidant defense. Guided by these DILI‐driven mechanisms, we develop a dual‐constraint deep‐learning model that jointly evaluates the interaction between therapeutic molecules and disease targets, enabling the identification of candidate molecules whose biological effects match the desired intervention. Through independent screening from FDA‐approved active pharmaceutical ingredients pool, we explore hesperidin (HES) and ursodeoxycholic acid (UDCA) as combination molecules capable of self‐assembling into uniform nanomedicines (HUNMs) with predicted biological activities. Flash nanocomplexation‐based engineering of HES and UDCA produces stable carrier‐free nanocrystals with improved aqueous dispersibility. In an acetaminophen‐challenged DILI mice, HUNMs alleviate hepatic injury, suppress inflammatory responses, restore glutathione homeostasis, and accelerate liver recovery. Together, our insights highlight an AI‐native strategy that harnesses smart molecules to develop a precise and translatable nanomedicine for efficient management of DILI and other complex diseases.

## Graphical abstract

An AI‐driven framework integrating disease‐network analysis, direction‐aware molecular screening, and carrier‐free co‐assembly engineering enables nanomedicine design. The dual‐constraint deep learning model predicts API–target affinity and regulatory direction to prioritize complementary APIs. Applied to drug‐induced liver injury, it identifies hesperidin and ursodeoxycholic acid, yielding carrier‐free nanomedicines with potent hepatoprotection and providing a generalizable discovery strategy.

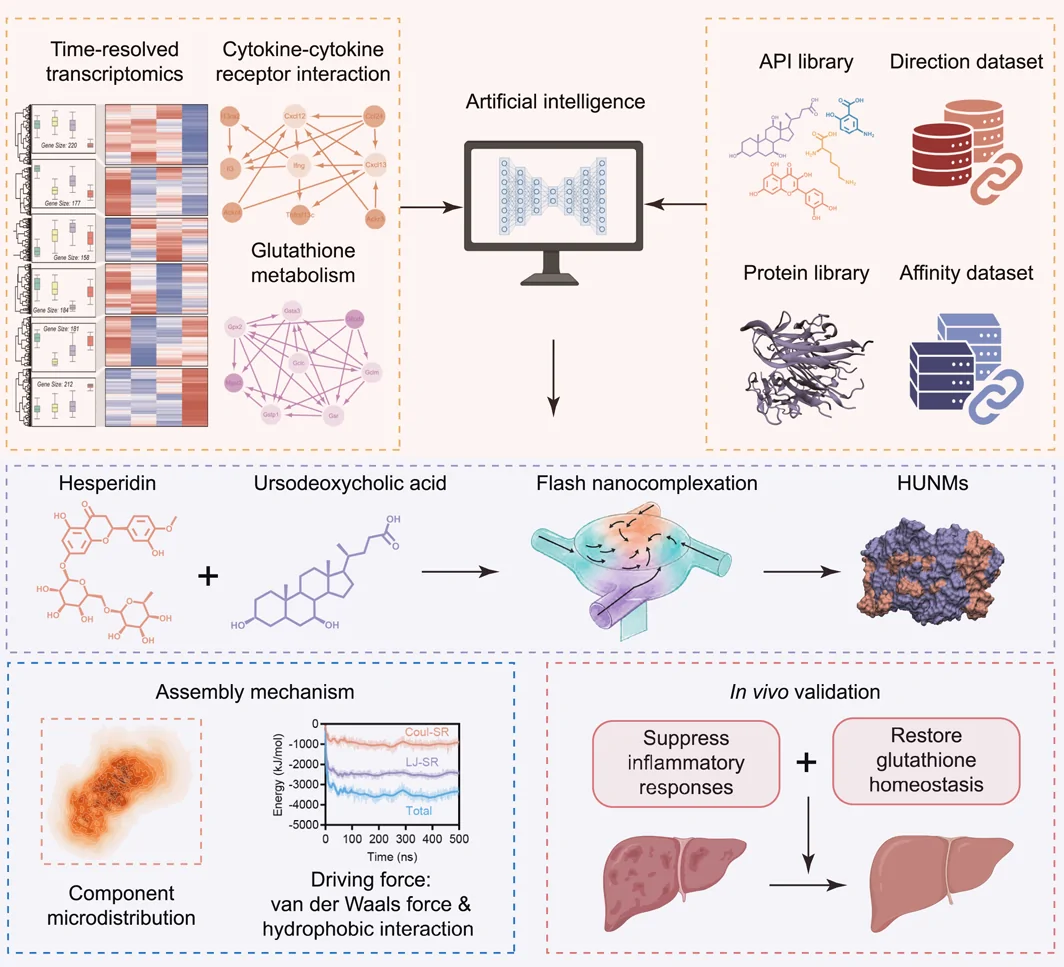

## 1 INTRODUCTION

Complex diseases are driven by coordinated perturbations of multiple pathological processes rather than by dysfunction of a single molecular target.[^1^, ^2^, ^3^] Drug‐induced liver injury (DILI), one of the leading causes of acute liver failure, epitomizes this complexity.[^4^, ^5^, ^6^, ^7^] Although diverse hepatotoxic insults initiate injury through distinct mechanisms, disease progression often converges on interconnected pathological programs involving inflammatory amplification, oxidative stress, and impaired tissue repair.[^8^, ^9^, ^10^] The existing anti‐oxidant therapies in clinic fail to modulate multiple pathological pathways concurrently. In this context, effective therapeutic intervention requires simultaneous modulation of multiple pathological pathways.[^11^, ^12^] Combination therapies offer an attractive strategy for addressing those multifactorial diseases, yet the rational development of active pharmaceutical ingredient (API)s that capable of regulating complementary disease processes remains a major challenge.[^13^, ^14^, ^15^]

Recent advances in artificial intelligence have accelerated API discovery through the prediction of API–target interactions.[^16^, ^17^, ^18^] Most existing approaches, however, prioritize target engagement without explicitly distinguishing whether a therapeutic molecule activates or inhibits its disease target.[^19^, ^20^, ^21^] Such an API–target interaction is decisive in complex diseases because of the therapeutic benefit depends not only on binding but also on steering biological networks toward a desired target.[^22^, ^23^] Moreover, disease‐relevant interventions are often temporally dependent, with distinct biological processes dominating different stages of disease progression. These considerations highlight the need for screening strategies capable of linking disease dynamics with sophisticated molecules that correspond to multiple pathological pathways.[^24^, ^25^, ^26^]

Although the screened APIs show therapeutic benefits in vitro and in vivo, most of them suffer from poor aqueous solubility and limited bioavailability, restricting their translational potential.[^27^, ^28^] Carrier‐free nanomedicines assembled entirely from APIs provide an appealing solution because they maximize drug loading while avoiding the complexity and safety concerns associated with synthetic carriers.[^29^, ^30^, ^31^] Yet successful co‐assembly contributes to the therapeutic outcomes more than they achieve pharmacological synergy. The constituent molecules must also possess complementary intermolecular interaction motifs capable of driving supramolecular organization through hydrogen bonding, π–π stacking, hydrophobic interactions, or electrostatic association. The exploration of API combinations that are biologically complementary and structurally compatible remains an unexplored design problem.[^32^, ^33^]

Here, we establish an integrated framework that links disease‐network analysis, direction‐aware molecular screening, and carrier‐free nanomedicine design. Time‐resolved transcriptomics is first employed to define mechanism‐specific therapeutics during disease progression and recovery. A dual‐constraint deep‐learning model is then developed to jointly evaluate API–target affinity and interaction type, enabling the identification of potent molecules whose biological effects match the desired intervention. Those candidates are subsequently screened for co‐assembly capability, thereby connecting therapeutic discovery with nanomedicine formulation.

We apply such an AI‐powered strategy in DILI management. Temporal transcriptomic analysis revealed two complementary repair axes during liver recovery: suppression of cytokine‐cytokine receptor signaling to attenuate inflammatory injury and activation of glutathione biosynthesis to restore antioxidant defense. Guided by these mechanistic insights, the dual‐constraint model independently identifies candidate molecules for each repair axis. Among the resulting combinations screened from FDA‐approved API pool, hesperidin (HES) and ursodeoxycholic acid (UDCA) are prioritized because they simultaneously exhibit the desired regulatory activities and favorable co‐assembly behavior. Flash nanocomplexation of engineering of HES and UDCA produces stable carrier‐free nanomedicines (HUNMs) with improved aqueous dispersibility.[^34^, ^35^, ^36^] In an acetaminophen‐challenged DILI moel, the sophisticated nanomedicines markedly alleviate hepatic injury, suppress inflammatory responses, restore glutathione homeostasis, and accelerate liver recovery, validating the coordinated modulation of both therapeutic axes predicted by the framework. Collectively, our work establishes a direction‐aware and chemistry‐guided framework that connects disease‐network interrogation with carrier‐free nanomedicine design, providing a generalizable route for discovering API combinations that integrate therapeutical synergy and structural adaptation for efficient and safe management of complex diseases.

## 2 RESULTS

### 2.1 Development of a dual‐constraint model for API‐target interaction prediction

**FIGURE 1:**
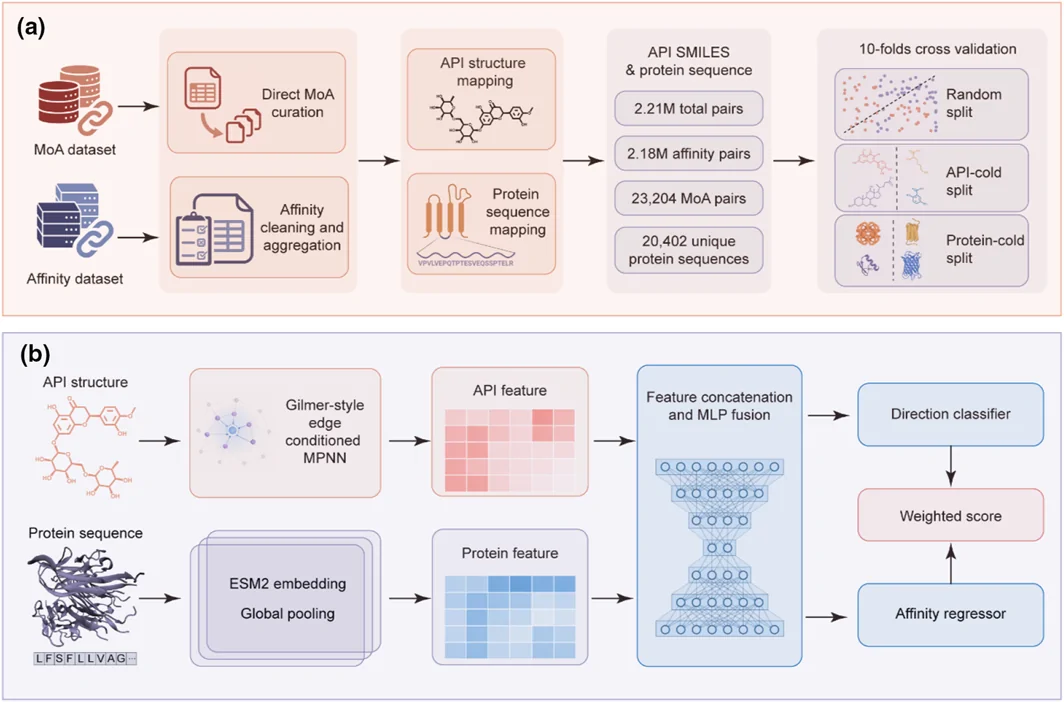
(a) Construction of the functional‐direction and affinity datasets. (b) General architecture of the evaluated models. API structures and protein sequences were independently encoded, fused and passed to task‐specific predictors.

To identify APIs with both strong target engagement and the desired regulatory direction, we constructed independent datasets for functional‐direction classification and affinity regression (Figure 1a). After chemical standardization, protein‐sequence mapping and pair‐level quality control, 23,204 API–protein pairs with explicit activating or inhibitory annotations were retained for classification, whereas approximately 2.18 million API–protein pairs were retained for affinity prediction. Three 10‐fold cross‐validation schemes were established to evaluate different generalization scenarios: pair‐random warm‐start splitting, protein cold‐start splitting based on 60% sequence identity, and API‐scaffold cold‐start splitting. Five representative API–protein architectures were benchmarked under identical training settings (Figure 1b).

**FIGURE 2:**
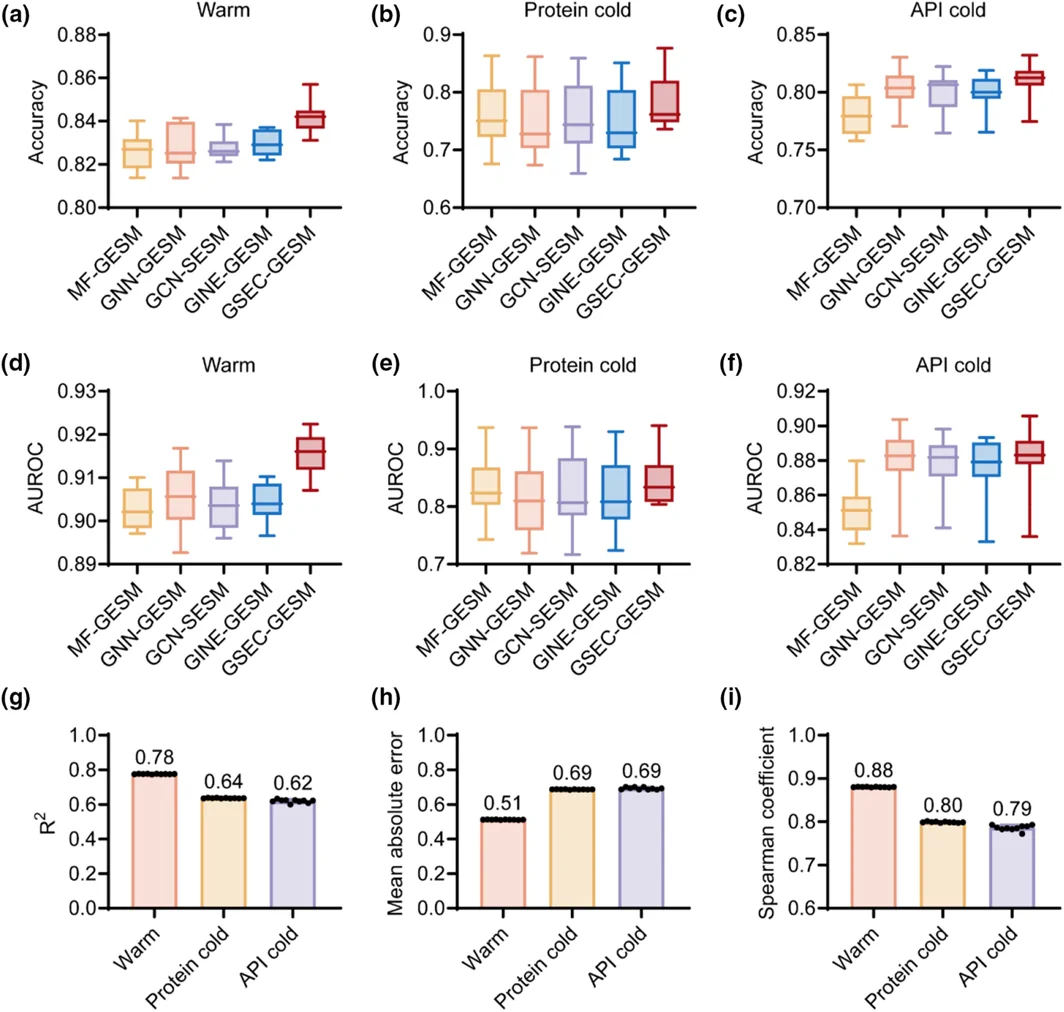
Classification accuracy under warm‐start (a), protein cold‐start (b) and API‐scaffold cold‐start conditions (c). AUROC under the corresponding warm‐start (d), protein cold‐start (e) and API‐scaffold cold‐start conditions (f). Coefficient of determination *R* ^2^ (g), MAE (h), and Spearman coefficient (i) of the GSEC–GESM model under warm‐start, protein cold‐start, and API cold‐start conditions. MF: a multilayer perceptron encoder based on 2048‐bit Morgan fingerprint; GCN, graph convolutional network; GINE, graph isomorphism network incorporating edge features; GNN, graph neural network; SESM, segment‐level Evolutionary Scale Modeling.

All models performed best under warm‐start conditions and showed reduced performance under protein‐ and API‐scaffold cold‐start evaluations, reflecting the increased difficulty of prediction in unseen biological or chemical spaces (Figure 2a–f). Among the tested architectures, Gilmer‐style edge conditioned message‐passing neural network (GSEC) combined with a global Evolutionary Scale Modeling (GESM) protein representation, achieved the best overall balance of classification performance and cross‐fold stability. Matthews Correlation Coefficient and area under precision‐recall curve (AUPRC) analyses yielded consistent conclusions (Supporting Information S1: Figure S1). GSEC–GESM was therefore selected as the final architecture for both functional‐direction classification and affinity regression. The affinity model showed the strongest performance under the warm‐start setting, with the highest R‐square and lowest mean absolute error (MAE), while retaining predictive capability under both protein and API cold‐start conditions (Figure 2g–i). The positive Spearman correlations across all three settings further indicated that the model preserved the relative ranking of binding affinities including previously unseen proteins and API scaffolds. Additional error‐based metrics yielded consistent results (Supporting Information S1: Figure S2). These results establish GSEC‐GESM as a reliable dual‐task architecture for independently predicting API regulatory direction and binding affinity under both conventional and cold‐start conditions.

### 2.2 Temporal transcriptomics identifies complementary repair programs in APAP‐induced liver injury

**FIGURE 3:**
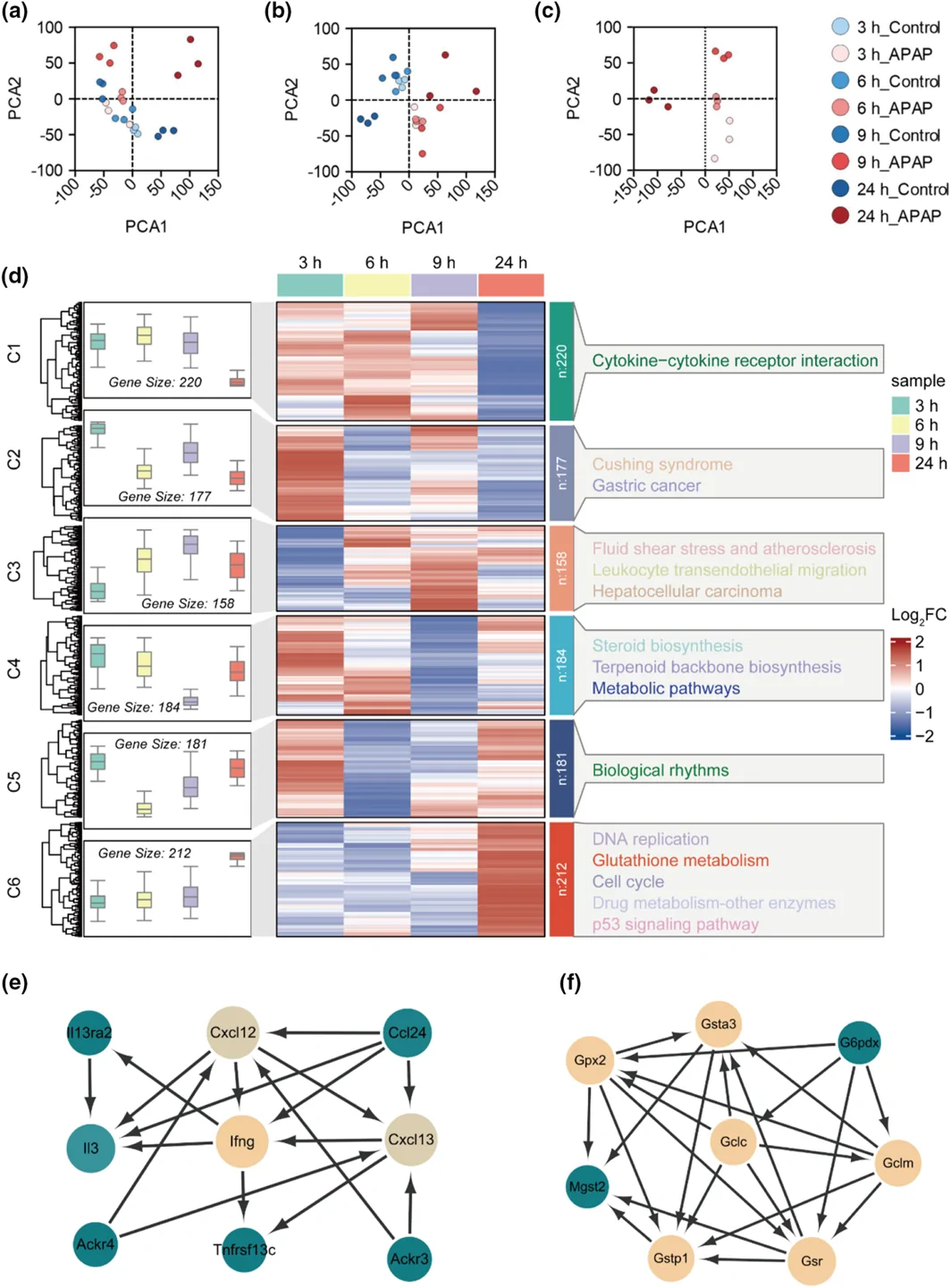
PCA of APAP‐treated and control samples before (a) and after correction for physiological temporal variation (b), and PCA of APAP‐treated samples across different time points after correction for physiological temporal variation (c). Pathway enrichment and expression patterns of differentially expressed genes across the APAP injury time course (d). Heatmap values represent the log_2_ FC in the APAP‐treated group relative to the corresponding control group, and pathways indicate the KEGG terms enriched by the respective gene sets. Protein–protein interaction networks of genes associated with cytokine–cytokine receptor interaction (e) and glutathione metabolism (f).

To identify therapeutic targets in DILI, we performed transcriptomic analysis of liver tissues collected at 3, 6, 9, and 24 h after high‐dose APAP administration. Principal component analysis showed clear separation between APAP‐treated rats and their controls after accounting for physiological temporal variation (Figure 3a,b). The APAP‐treated groups also showed a clear time‐dependent pattern, with gradual changes from 3 to 9 h and a distinct transcriptional profile emerging at 24 h (Figure 3c). This pattern was further supported by the intergroup correlation matrix, in which the 24 h APAP group showed the greatest divergence from the earlier time points (Supporting Information S1: Figure S3). Pathway analysis of differentially expressed genes further revealed a transition between the early injury and late recovery phases (Figure 3d and Supporting Information S1: Figure S4). At 24 h, genes enriched in the cytokine–cytokine receptor interaction pathway were broadly downregulated, suggesting that the liver had shifted toward an anti‐inflammatory phenotype. In parallel, genes involved in glutathione metabolism were upregulated, indicating recovery of antioxidant capacity (Figure 3e,f). These results suggest that the liver entered a repair‐associated transcriptional state at 24 h, characterized by inflammatory suppression and recovery of glutathione‐dependent antioxidant defenses. These distinct changes in gene expression defined two complementary therapeutic directions for subsequent API screening in DILI.

### 2.3 AI‐guided prioritization and engineering of hesperidin‐ursodeoxycholic acid nanomedicine

**FIGURE 4:**
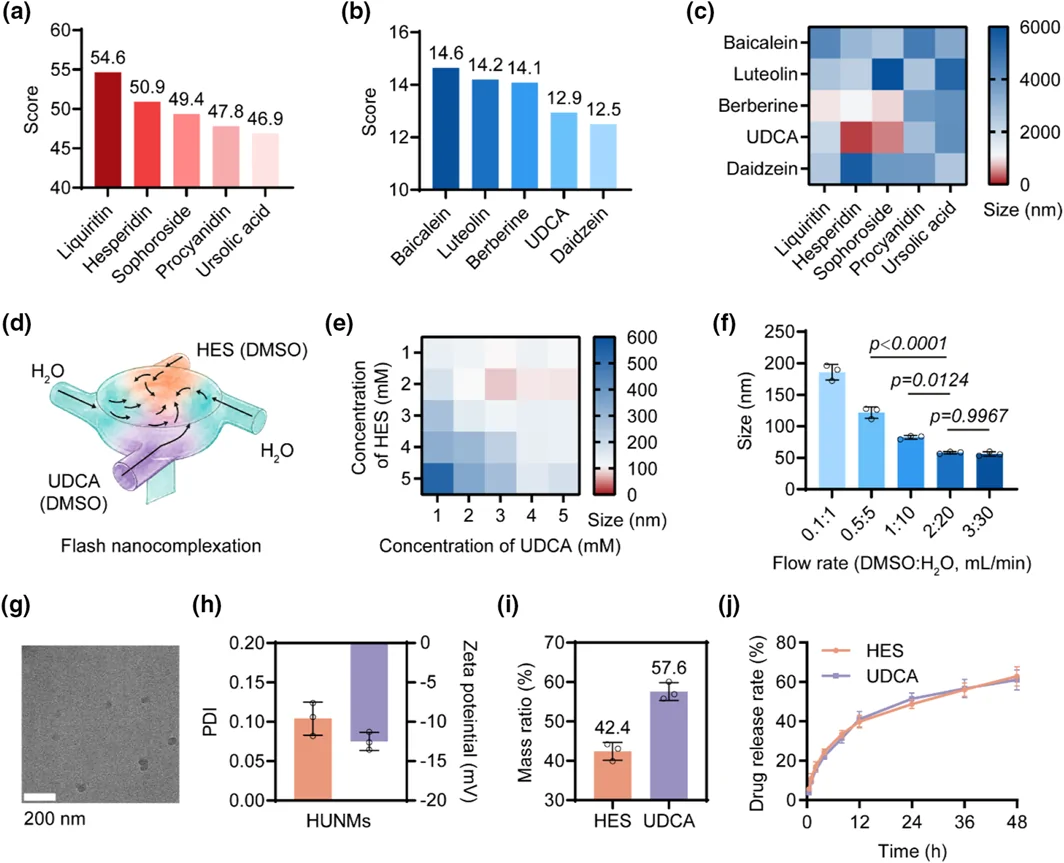
The top‐5 APIs for inhibition of genes associated with the cytokine–cytokine receptor interaction pathway (a), and activation of genes associated with the glutathione metabolism pathway (b). Hydrodynamic diameters of the co‐assembled products formed by pairwise combinations of the top‐ranked candidates from the two therapeutic directions (c). Schematic illustration of HUNM preparation by flash nanocomplexation (d). Particle sizes of co‐assembled products prepared using different HES and UDCA concentrations (e), and particle sizes of HUNMs prepared at different flow rates (f). Transmission electron microscopy (TEM) image (g), polydispersity index (PDI), and zeta potential of the optimized HUNMs (h). Relative contents of HES and UDCA in HUNMs (i), and in vitro release profiles of HES and UDCA in phosphate‐buffered saline (PBS) (j).

The differentially expressed genes enriched in the cytokine–cytokine receptor interaction pathway and glutathione metabolism pathway were used as two independent target sets for API screening. More than 10,000 natural bioactive compounds were evaluated using the GSEC–GESM models. Liquiritin, hesperidin (HES), sophoroside, procyanidin, and ursolic acid showed the highest integrated scores for inhibition of cytokine‐associated targets (Figure 4a and Supporting Information S1: Figure S5), whereas baicalein, luteolin, berberine, ursodeoxycholic acid (UDCA), and daidzein ranked highest for activation of glutathione metabolism‐associated targets (Figure 4b and Supporting Information S1: Figure S6). Most of these candidates exhibit limited aqueous solubility, which typically results in oral bioavailability. The top‐ranked molecules from the two screening directions were then paired to evaluate their ability to co‐assemble into nanomedicine. Among the tested combinations, HES and UDCA produced the smallest and most uniform particles and were therefore selected for further formulation development (Figure 4c).

The formulation composition and mixing conditions for the flash nanocomplexation‐based preparation of HES–UDCA nanomedicines (HUNMs) were systematically optimized (Figure 4d). Among the tested formulation compositions, a HES‐to‐UDCA concentration ratio of 2–3 mM yielded the smallest particle size (Figure 4e). Under this formulation, increasing the aqueous flow rate progressively decreased the particle size until reaching a plateau at 20 ml min^−1^, which was therefore selected as the optimal mixing condition for subsequent preparation (Figure 4f). The resulting HUNMs exhibited a mean hydrodynamic diameter of 58.17 ± 1.76 nm, a polydispersity index (PDI) of 0.104 ± 0.021, and a ζ‐potential of −12.48 ± 1.14 mV. HES and UDCA accounted for 42.4% and 57.6% of the API content, respectively (Figure 4g–i). The X‐ray diffraction patterns of HUNMs exhibited broadened, shifted, and redistributed diffraction peaks relative to the physical mixture, suggesting reduced crystallite dimensions and reorganization of molecular packing during nanoparticle formation (Supporting Information S1: Figure S7). The stability of HUNMs under simulated physiological conditions was further evaluated. HUNMs exhibited only a slight initial increase in particle size and minimal changes in PDI during incubation in different media (Supporting Information S1: Figure S8). In physiological buffer, approximately 50% of both components were released within 24 h, indicating sustained drug release from the co‐assembled nanomedicine (Figure 4j).

### 2.4 Molecular dynamics simulation reveals the co‐assembly mechanism of HES and UDCA

**FIGURE 5:**
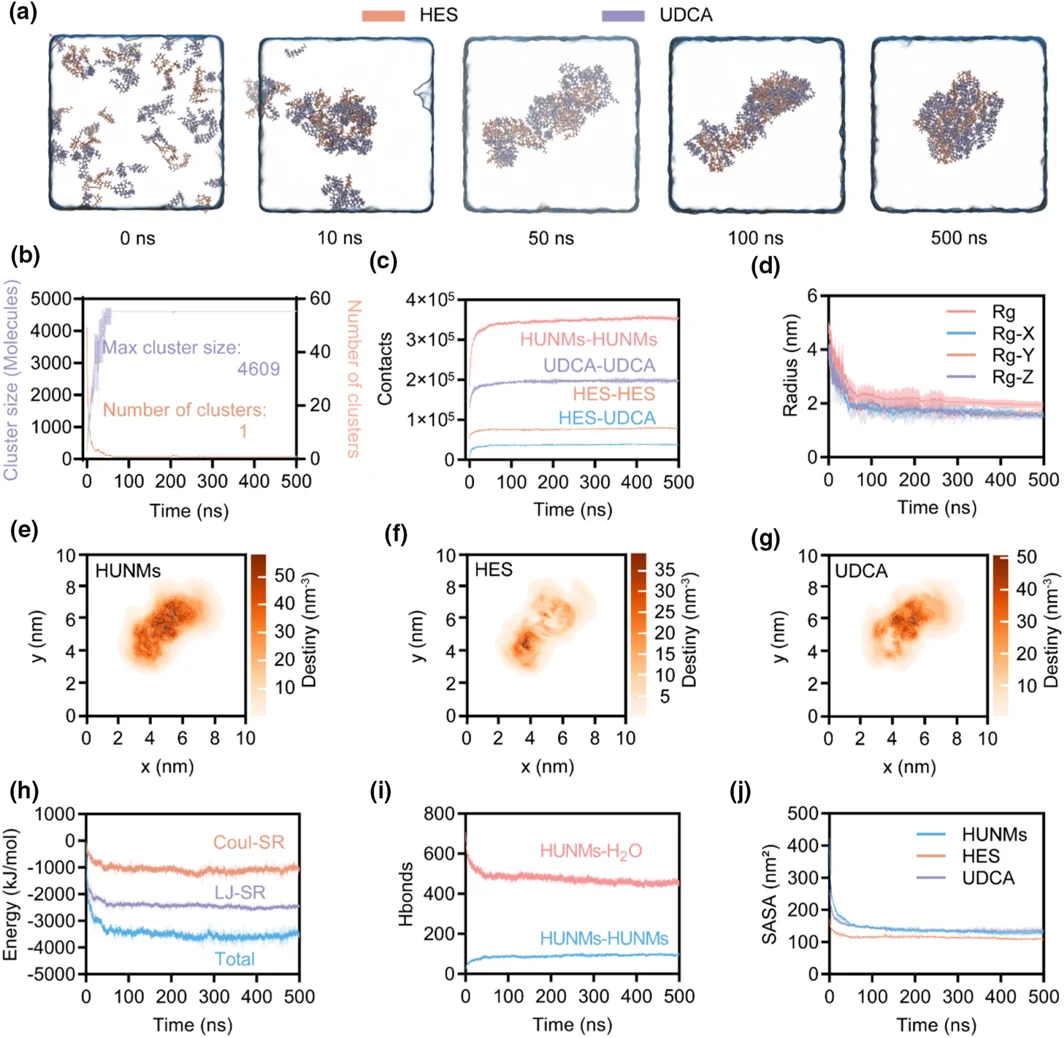
Snapshots of the HES–UDCA assembly process during molecular dynamics simulation (a). Time‐dependent changes in number of clsuters and max cluster size (b), the number of close‐contact atomic pairs (c), and the overall radius of gyration of the cluster (d). Two‐dimensional density projection maps of the final cluster showing the overall distribution (e), the HES distribution (f), and the UDCA distribution (g). Time‐dependent changes in intermolecular interaction energy between HES and UDCA (h), the numbers of hydrogen bonds within the cluster and between the cluster and water molecules (i), and the solvent‐accessible surface area (SASA) of the cluster (j). In (b–d) and (h–j), the solid curves represent the average values obtained from three independent simulations, and the shaded regions represent the corresponding standard deviation (SD) calculated from these simulations.

To further understand the formation mechanism of HUNMs, we performed molecular dynamics simulations of the co‐assembly process of HES and UDCA in water. HES and UDCA underwent rapid aggregation during the initial stage of simulation, characterized by a rapid decrease in cluster number and increase in cluster size within the first 10 ns (Figure 5a–c and Supporting Information S1: Figures S9 and S10). The initially formed small clusters subsequently coalesced into a dominant large cluster at approximately 50 ns, followed by gradual structural rearrangement and stabilization. The structural parameters entered a slow‐evolution stage and reached a relatively stable state at approximately 300 ns. The radii of gyration along the three principal axes were comparable, indicating an approximately isotropic cluster morphology (Figure 5d). Density projection analyses further revealed nonuniform molecular distributions within the final cluster. Both one‐dimensional and two‐dimensional density maps showed partial segregation of HES and UDCA, suggesting a certain degree of self‐association of the same molecular species within the co‐assembled structure (Figure 5e–g and Supporting Information S1: Figure S11).

Analysis of the intermolecular interaction energies showed that the short‐range (SR) Lennard–Jones (LJ) term was dominant throughout the assembly process (Figure 5h), indicating that van der Waals interactions were parts of the major driving force for cluster formation. In contrast, only limited hydrogen bonding was observed within the cluster itself, whereas abundant hydrogen bonds were detected between the cluster and surrounding water molecules (Figure 5i). Combined with the time‐dependent solvent‐accessible surface area and the distributions of molecular electrostatic potential and hydrophobicity (Figure 5j, Supporting Information S1: Figures S12 and S13), these results suggest that van der Waals and hydrophobic interactions primarily drove the HES–UDCA co‐assembly, while hydrogen bonding between the cluster surface and water contributed to the stable dispersion of HUNMs in aqueous solution.

### 2.5 HUNMs improve cellular uptake and protect against inflammatory and oxidative injury

**FIGURE 6:**
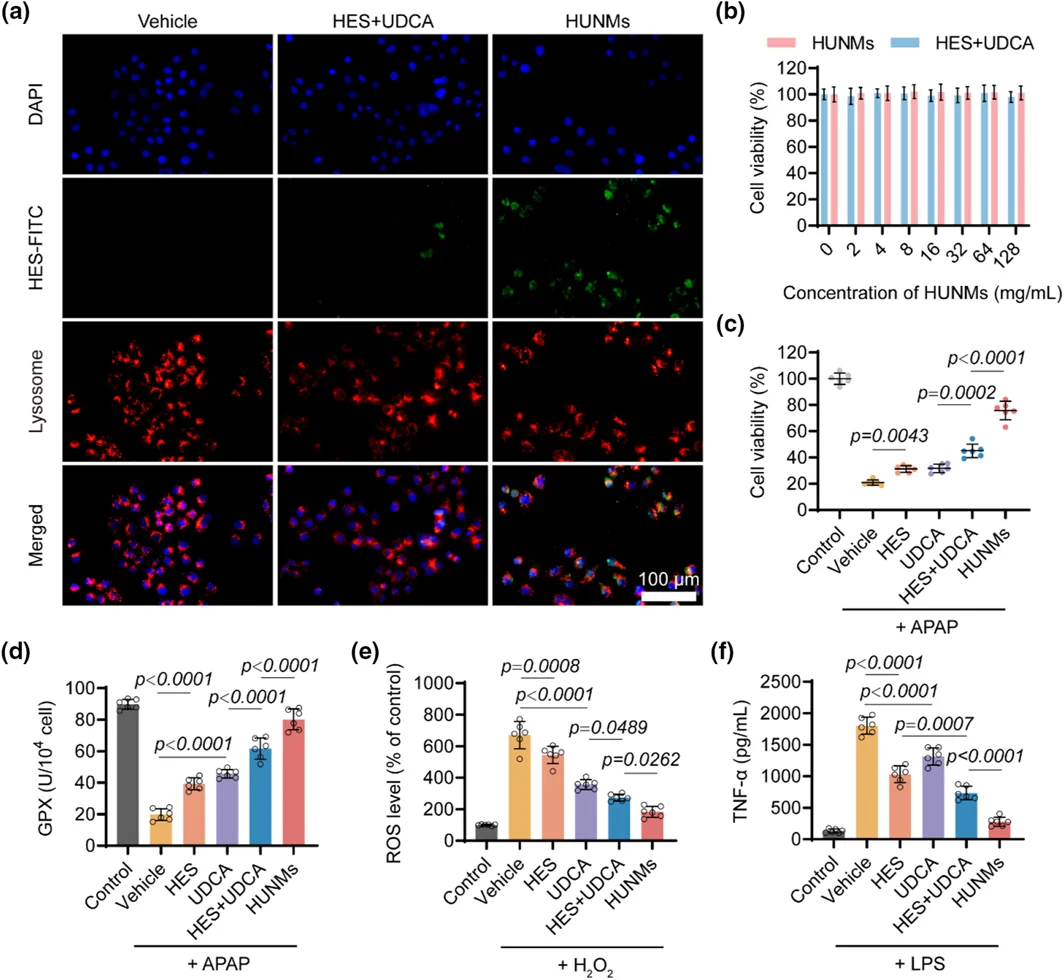
Cellular uptake of FITC‐labeled free HES and FITC‐labeled HUNMs after 2 h of incubation (a). Cell viability after 24 h incubation with HUNMs or an equivalent dose of free HES + UDCA (b). Protective effects of free HES, free UDCA, the free HES + UDCA combination, and HUNMs against APAP‐induced cell injury (c). Recovery of GPX activity in the APAP‐induced cell‐injury model after treatment with free HES, free UDCA, the free HES + UDCA combination, or HUNMs (d). Intracellular ROS levels in the H_2_O_2_‐induced oxidative‐stress model after treatment (e). Inhibitory effect of free HES, free UDCA, the free HES + UDCA combination, and HUNMs on TNF‐α production (f). ROS, reactive oxygen species.

We next evaluated the cellular uptake of HUNMs using FITC‐labeled HES. After 2 h of incubation, cells treated with HUNMs exhibited markedly stronger intracellular fluorescence than those treated with the free HES–UDCA combination, indicating more efficient cellular internalization of the co‐assembled nanomedicine (Figure 6a and Supporting Information S1: Figure S14). Notably, neither formulation‐induced appreciable cytotoxicity after 24 h of incubation, even at medium and high concentrations (Figure 6b). The hepatoprotective activity of HUNMs was subsequently assessed in an APAP‐induced hepatocyte injury model. Compared with the free drug combination, HUNMs more effectively restored cell viability following APAP exposure (Figure 6c). This enhanced protection was accompanied by a greater recovery of glutathione peroxidase (GPX) activity (Figure 6d), indicating improved restoration of cellular antioxidant capacity.

To further validate these bioactivities, HUNMs were evaluated in independent models of oxidative stress and inflammation. In H_2_O_2_‐treated HepG2 cells, HUNMs substantially reduced intracellular reactive oxygen species accumulation (Figure 6e). Likewise, in LPS‐stimulated RAW264.7 macrophages, HUNMs significantly decreased tumor necrosis factor‐α (TNF‐α) secretion compared with the free API combination (Figure 6f). Collectively, these findings demonstrate that co‐assembly into HUNMs enhances intracellular delivery, thereby conferring superior hepatoprotective, antioxidant, and anti‐inflammatory activities.

### 2.6 In vivo therapeutic efficacy of HUNMs

**FIGURE 7:**
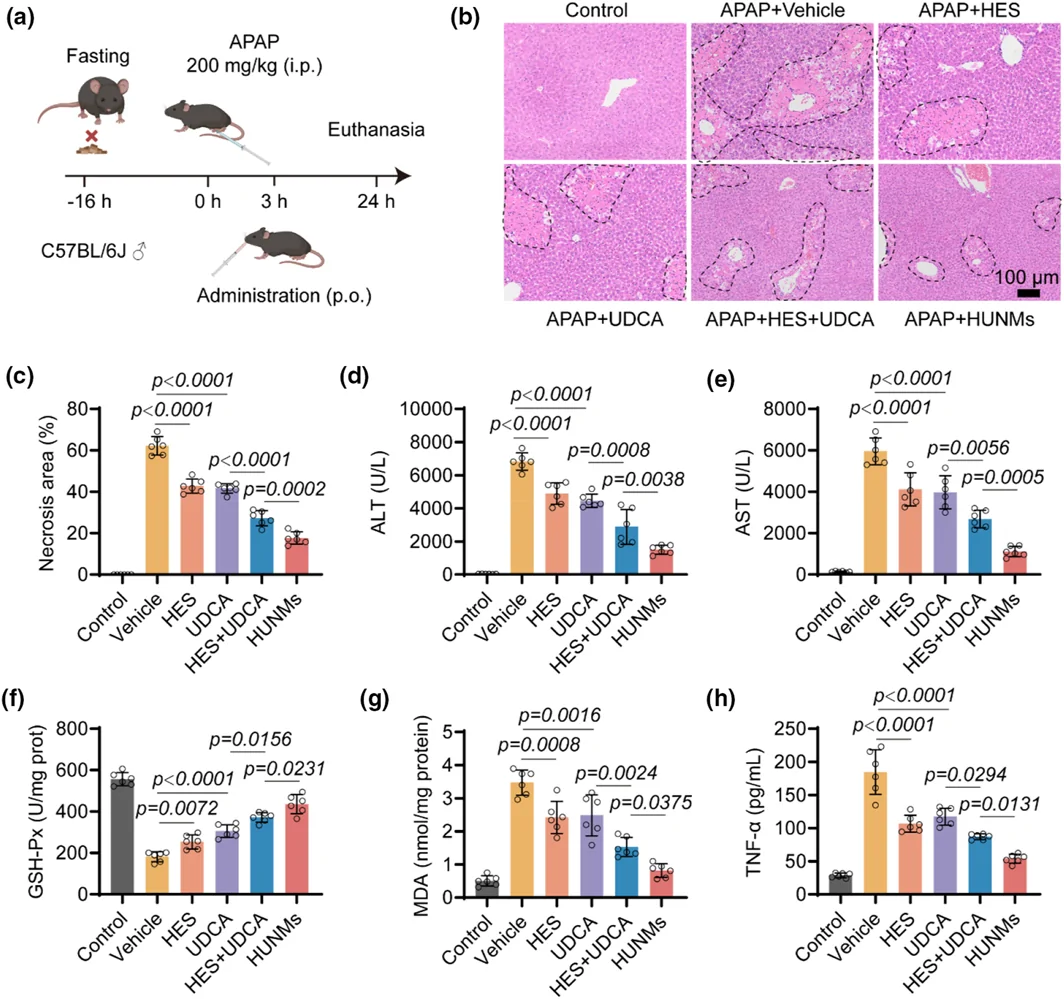
Schematic illustration of APAP‐induced liver injury and the treatment protocol in mice (a). Representative H&E‐stained liver sections from different groups at 24 h after APAP administration (b). Quantification of liver necrosis area (c), serum ALT levels (d), serum AST levels (e), hepatic glutathione peroxidase activity (f), hepatic malondialdehyde (MDA) content (g), and hepatic TNF‐α levels (h) at 24 h after modeling.

In an APAP‐induced liver injury mouse model, therapeutic intervention administered after injury onset produced pronounced hepatoprotective effects. Relative to both the injury and free API groups, HUNM treatment substantially reduced hepatic necrotic regions (Figure 7a–c). Systemically, liver damage was alleviated, as evidenced by decreased serum alanine aminotransferase (ALT) and aspartate aminotransferase (AST) levels (Figure 7d,e). In addition, oxidative stress was effectively mitigated, as reflected by the recovery of glutathione peroxidase (GPX) activity and diminished malondialdehyde (MDA) accumulation (Figure 7f,g), together with a significant reduction in TNF‐α, indicating suppression of inflammatory signaling (Figure 7h). These findings indicate that HUNMs counteract APAP‐induced hepatotoxicity through coordinated regulation of inflammatory and redox‐related processes, in agreement with the dual‐pathway mechanisms identified in the systems‐level analysis.

## 3 CONCLUSION

In summary, we establish an AI‐driven framework for nanomedicine design that integrates disease‐network analysis, direction‐aware molecular screening, and carrier‐free co‐assembly engineering for complex diseases. A dual‐constraint deep learning architecture (GSEC–GESM) was developed to jointly predict API–target binding affinity and regulatory direction, enabling function‐guided molecular prioritization with strong generalization across unseen proteins and chemical scaffolds. Applied to DILI, time‐resolved transcriptomics delineates two coordinated pathological axes involving inflammatory cytokine signaling and glutathione‐mediated redox imbalance, which define the design principles for therapeutic intervention. Guided by these mechanisms, hesperidin (HES) and ursodeoxycholic acid (UDCA) are identified as a complementary pair with matched biological functions and intrinsic co‐assembly compatibility. Molecular dynamics simulations and flash nanocomplexation confirm their spontaneous assembly into stable carrier‐free nanomedicines (HUNMs) with enhanced cellular delivery. In vivo, HUNMs effectively mitigate APAP‐induced hepatotoxicity by suppressing inflammation and oxidative stress while restoring liver function. Overall, this work presents an AI‐enabled, direction‐aware nanomedicine discovery paradigm that connects systems‐level disease understanding with rational API co‐assembly for precision therapy of multifactorial diseases.

## 4 EXPERIMENTAL SECTION/METHODS

### 4.1 Dataset construction

Chemical–protein interaction records from Comparative Toxicogenomics Database were standardized at the API–protein pair level, and entries with invalid structures, unresolved identities, unmapped sequences, or conflicting annotations were removed.^[37]^ Pairs with explicit activating or inhibitory effects were retained for classification, yielding 23,204 samples. Affinity measurements were harmonized, aggregated, and filtered to obtain approximately 2.18 million nonconflicting pairs from BindingDB and PDBbind v2016.[^38^, ^39^]

### 4.2 Model development and evaluation

Five API–protein architectures were compared under identical training settings: a multilayer perceptron (MLP) applied to 2048‐bit Morgan fingerprints, a graph neural network, a graph isomorphism network incorporating edge features, and a Gilmer‐style edge‐conditioned message‐passing molecular encoder (GSEC), each paired with a GESM protein representation (GESM), as well as a graph convolutional molecular encoder paired with segment‐level ESM protein representations. The selected GSEC–GESM model combined a Gilmer‐style edge conditioned message‐passing molecular encoder with a global ESM protein representation and feature concatenation.[^40^, ^41^, ^42^, ^43^] API embeddings generated by the API encoder were aggregated using masked mean pooling and concatenated with the protein embedding. The concatenated features were processed by a MLP fusion module to generate a unified API–protein interaction representation. For each task, this representation was passed to an MLP classifier producing activity‐direction prediction, or an MLP regressor generating a single continuous output for affinity prediction. The activity‐direction classifier and affinity regressor adopted the same input representations and encoder–fusion architecture but were instantiated and trained as two independent models. Performance was evaluated using 10‐fold warm‐start, protein cold‐start, and API‐scaffold cold‐start cross‐validation. Classification was assessed using accuracy, Matthews Correlation Coefficient, AUROC, and AUPRC, whereas regression was evaluated using *R* ^2^, MAE, RMSE, MSE, median absolute error, and Pearson and Spearman correlations.

### 4.3 Transcriptomic analysis

Rat liver transcriptomic data were obtained from Open TG‐GATEs using the high‐dose APAP groups and their corresponding time‐matched controls at 3, 6, 9, and 24 h.^[44]^ Time‐matched control profiles were used to account for physiological temporal variation. Principal component analysis, intergroup correlation analysis, and differential expression analysis were performed using Python. Differentially expressed genes were defined using an absolute log_2_ (fold change) greater than 1 and a *p* value below 0.05. KEGG pathway enrichment and protein–protein interaction network analyses were conducted using the STRING platform.[^45^, ^46^] Genes enriched in cytokine–cytokine receptor interaction and glutathione metabolism were subsequently used as intervention targets for API screening.

### 4.4 Computational simulations

The self‐assembly behavior of Hesperidin (HES) and Ursodeoxycholic acid (UDCA) was investigated using all‐atom molecular dynamics simulations implemented in GROMACS 2025.2.^[47]^ Atomic partial charges were assigned using the RESP2 scheme based on quantum chemical calculations performed with ORCA and processed using Multiwfn.[^48^, ^49^, ^50^] Molecular topologies were generated with Sobtop employing the General Amber Force Field, and the system constructed by randomly placing 21 molecules of HES and 44 molecules of UDCA in a cubic box of 10 × 10 × 10 nm was solvated with TIP3P water.[^51^, ^52^] Energy minimization was performed using the steepest‐descent method, after which the system was equilibrated for 100 ps under NVT ensemble and subsequently for 100 ps under NPT ensemble. Temperature and pressure were maintained at 300 K and 1 bar using the V‐rescale thermostat and Parrinello–Rahman barostat, respectively. A 500 ns production trajectory was then generated with a 2 fs time step. The resulting trajectories were analyzed to characterize cluster evolution, structural organization, and intermolecular interaction energies, providing mechanistic insights into the molecular forces driving HUNM self‐assembly. The simulation procedure was independently repeated three times.

### 4.5 Cellular uptake and cytotoxicity

HepG2 cells were cultured in Dulbecco's modified Eagle's medium supplemented with 10% fetal bovine serum and 1% penicillin–streptomycin. For cellular uptake studies, cells were incubated with FITC‐labeled HES or HUNMs at an equivalent FITC‐labeled HES concentration of 25 μg ml^−1^ for 2 h. Lysosomes and nuclei were subsequently stained with LysoTracker Red (75 nM) and DAPI (0.1 μg ml^−1^), respectively. The cells were washed three times with phosphate‐buffered saline (PBS) to remove free fluorescent molecules and imaged using a fluorescence microscope. The fluorescence‐positive areas corresponding to HES‐FITC signals and nuclei were quantified using ImageJ software. The normalized fluorescence was calculated by normalizing the HES‐FITC fluorescence area to the blue fluorescence area of nuclei. Cytotoxicity was evaluated using a Cell Counting Kit‐8 (CCK‐8) assay following the manufacturer's protocol.

### 4.6 In vitro evaluation of hepatoprotective activity

Acetaminophen (APAP)‐induced hepatocyte injury model was established by exposing HepG2 cells to 5 mM APAP for 2 h, followed by incubation with free HES, free UDCA, a physical mixture of HES and UDCA, or HUNMs for 24 h at an equivalent HES concentration of 42.4 μg ml^−1^. Cell viability was determined using a CCK‐8 assay, and glutathione peroxidase (GPX) activity was measured with a commercial assay kit.

### 4.7 Animal model

8‐week‐old male C57BL/6 mice were supplied by GemPharmatech and maintained under specific pathogen‐free conditions. After a 1‐week acclimation period, animals were randomly allocated into six experimental groups (*n* = 6): Control, Vehicle, HES, UDCA, HES + UDCA, and HUNMs. Acute liver injury was induced by intraperitoneal injection of acetaminophen (200 mg kg^−1^) after 16 h of fasting, while control mice received an equal volume of PBS. Therapeutic intervention was initiated 3 h later by oral gavage of HES, UDCA, HES + UDCA, or HUNMs at an equivalent HES dose of 50 mg kg^−1^ 24 hours after APAP injection, blood and liver specimens were collected for subsequent evaluation.

### 4.8 Histological and biochemical analyses

Liver function was assessed by measuring serum alanine aminotransferase (ALT) and aspartate aminotransferase (AST), whereas the inflammatory response was evaluated by quantifying TNF‐α using ELISA. Hepatic glutathione peroxidase (GPX) activity and malondialdehyde (MDA) levels were determined using commercial assay kits. For morphological assessment, liver samples were fixed in 4% paraformaldehyde, embedded in paraffin, sectioned, and subjected to hematoxylin and eosin (H&E) staining. The extent of liver injury was quantified as the percentage of necrotic areas using ImageJ.

### 4.9 Statistical analysis

Data are presented as mean ± SD. Group comparisons were performed using one‐way ANOVA with Tukey's post hoc test. For model comparisons, box plots represent the full range of the data (minimum to maximum).

## CONFLICT OF INTEREST STATEMENT

The authors declare no conflicts of interest.

## ETHICS STATEMENT

The study procedures were approved by the Ethical Committee of the West China Hospital, Sichuan University (20260331017).

## Supporting information

Supporting Information S1

## Data Availability

The data that support the findings of this study are available from the corresponding author upon reasonable request. The source code is available via GitHub at https://github.com/Ilsazive‐Ruo/DTI.git.

## References

[1] J. X. Hu , C. E. Thomas , S. Brunak , Nat. Rev. Genet. 2016, 17, 615. 27498692 10.1038/nrg.2016.87

[2] D. E. Cameron , C. J. Bashor , J. J. Collins , Nat. Rev. Microbiol. 2014, 12, 381. 24686414 10.1038/nrmicro3239

[3] F. J. Bruggeman , H. V. Westerhoff , Trends Microbiol. 2007, 15, 45. 17113776 10.1016/j.tim.2006.11.003

[4] M. Sun , P. Chen , K. Xiao , X. Zhu , Z. Zhao , C. Guo , X. He , T. Shi , Q. Zhong , Y. Jia , Y. Tao , M. Li , K. W. Leong , D. Shao , Adv. Sci. 2023, 10, 2206789. 10.1002/advs.202206789 PMC10238175 37035952

[5] H. Jaeschke , A. Ramachandran , Annu. Rev. Pathol. Mech. Dis. 2024, 19, 453. 10.1146/annurev-pathmechdis-051122-094016 PMC11131139 38265880

[6] R. J. Andrade , N. Chalasani , E. S. Björnsson , A. Suzuki , G. A. Kullak‐Ublick , P. B. Watkins , H. Devarbhavi , M. Merz , M. I. Lucena , N. Kaplowitz , G. P. Aithal , Nat. Rev. Dis. Primers 2019, 5, 58. 31439850 10.1038/s41572-019-0105-0

[7] M. Chen , A. Suzuki , J. Borlak , R. J. Andrade , M. I. Lucena , J. Hepatol. 2015, 63, 503. 25912521 10.1016/j.jhep.2015.04.016

[8] M. P. Holt , C. Ju , AAPS J. 2006, 8, 6. 10.1208/aapsj080106 PMC2751423 16584133

[9] R. Allison , A. Guraka , I. T. Shawa , G. Tripathi , W. Moritz , A. Kermanizadeh , J. Toxicol. Environ. Health B 2023, 26, 442. 10.1080/10937404.2023.2261848 37786264

[10] C. E. Goldring , G. Russomanno , C. Pin , P. Trairatphisan , K. A. Beattie , C. P. Fisher , J. Piñero , R. J. Brennan , D. Clausznitzer , I. M. Copple , T. M. de Kok , C. A. Duckworth , L. I. Furlong , B. Füzi , A. Gabor , L. Gall , J. Hengstler , H. Hermjakob , F. Hunter , D. Jennen , M. Koskinen , S. J. Kunnen , L. Lammens , S. Lobentanzer , M. Mohr , E. Passini , D. M. Pritchard , R. S. Malik‐Sheriff , B. Rodríguez , E. I. Rossman , J. Saez‐Rodríguez , F. Schmidt , R. Sison‐Young , I. Soininen , S. Turner , B. van de Water , J. G. C. van Hasselt , F. Venezia , J. A. Willy , D. J. Leishman , J. L. Stevens , L. Laplanche , Nat. Rev. Drug Discov. 2026, 25, 138. 41145622 10.1038/s41573-025-01308-z

[11] F. Vincent , A. Nueda , J. Lee , M. Schenone , M. Prunotto , M. Mercola , Nat. Rev. Drug Discov. 2022, 21, 899. 35637317 10.1038/s41573-022-00472-w PMC9708951

[12] T. Haikarainen , A. T. Virtanen , B. F. Cravatt , O. Silvennoinen , Nat. Rev. Drug Discov. 2026, 25, 268. 41486192 10.1038/s41573-025-01336-9

[13] R. Bayat Mokhtari , T. S. Homayouni , N. Baluch , E. Morgatskaya , S. Kumar , B. Das , H. Yeger , Oncotarget 2017, 8, 38022. 28410237 10.18632/oncotarget.16723 PMC5514969

[14] J. B. Fitzgerald , B. Schoeberl , U. B. Nielsen , P. K. Sorger , Nat. Chem. Biol. 2006, 2, 458. 16921358 10.1038/nchembio817

[15] S. K. Sarin , A. Choudhury , A. Kumar , N. Mahmud , G. H. Lee , Q. Ning , S.‐S. Tan , K. Thanapirom , V. Arora , N. Nakayama , J. Li , C. J. Karvellas , Nat. Rev. Gastroenterol. Hepatol. 2026, 23, 411. 41486194 10.1038/s41575-025-01159-4

[16] X. Chen , C. C. Yan , X. Zhang , X. Zhang , F. Dai , J. Yin , Y. Zhang , Brief. Bioinform. 2016, 17, 696. 26283676 10.1093/bib/bbv066

[17] Z. Tanoli , A. Fernández‐Torras , U. O. Özcan , A. Kushnir , K. M. Nader , Y. Gadiya , L. Fiorenza , A. Ianevski , M. Vähä‐Koskela , M. Miihkinen , U. Seemab , H. Leinonen , B. Seashore‐Ludlow , M. Tampere , A. Kalman , F. Ballante , E. Benfenati , G. Saunders , S. Potdar , I. Gómez García , R. García‐Serna , C. Talarico , A. R. Beccari , W. Schaal , A. Polo , S. Costantini , E. Cabri , M. Jacobs , J. Saarela , A. Budillon , O. Spjuth , P. Östling , H. Xhaard , J. Quintana , J. Mestres , P. Gribbon , A. E. Ussi , D. C. Lo , M. de Kort , K. Wennerberg , M. Fratelli , J. Carreras‐Puigvert , T. Aittokallio , Nat. Rev. Drug Discov. 2025, 24, 521. 40102635 10.1038/s41573-025-01164-x

[18] J. Abramson , J. Adler , J. Dunger , R. Evans , T. Green , A. Pritzel , O. Ronneberger , L. Willmore , A. J. Ballard , J. Bambrick , S. W. Bodenstein , D. A. Evans , C.‐C. Hung , M. O’Neill , D. Reiman , K. Tunyasuvunakool , Z. Wu , A. Žemgulytė , E. Arvaniti , C. Beattie , O. Bertolli , A. Bridgland , A. Cherepanov , M. Congreve , A. I. Cowen‐Rivers , A. Cowie , M. Figurnov , F. B. Fuchs , H. Gladman , R. Jain , Y. A. Khan , C. M. R. Low , K. Perlin , A. Potapenko , P. Savy , S. Singh , A. Stecula , A. Thillaisundaram , C. Tong , S. Yakneen , E. D. Zhong , M. Zielinski , A. Žídek , V. Bapst , P. Kohli , M. Jaderberg , D. Hassabis , J. M. Jumper , Nature 2024, 630, 493. 38718835 10.1038/s41586-024-07487-w PMC11168924

[19] F. W. Pun , D. Podolskiy , E. Izumchenko , A. Mortlock , T. I. Oprea , M. Scheibye‐Knudsen , K. Fortney , E. Morgen , F. Ren , A. Zhavoronkov , Nat. Rev. Drug Discov. 2026, 25, 534. 42009769 10.1038/s41573-026-01412-8

[20] R. Chen , Á. Duffy , R. Do , Nat. Rev. Genet. 2026, 27, 231. 41198831 10.1038/s41576-025-00904-4 PMC12816132

[21] P. Bai , F. Miljković , B. John , H. Lu , Nat. Mach. Intell. 2023, 5, 126.

[22] M. Bagherian , E. Sabeti , K. Wang , M. A. Sartor , Z. Nikolovska‐Coleska , K. Najarian , Brief. Bioinform. 2021, 22, 247. 31950972 10.1093/bib/bbz157 PMC7820849

[23] W. Chen , X. Liu , S. Zhang , S. Chen , Mol. Ther. Nucleic Acids 2023, 31, 691. 36923950 10.1016/j.omtn.2023.02.019 PMC10009646

[24] D. S. Fischer , M. A. Villanueva , P. S. Winter , A. K. Shalek , Nat. Rev. Genet. 2025, 26, 514. 40065155 10.1038/s41576-025-00821-6 PMC13552703

[25] L. Qiao , A. Khalilimeybodi , N. J. Linden‐Santangeli , P. Rangamani , Annu. Rev. Biomed. Eng. 2025, 27, 425. 39971380 10.1146/annurev-bioeng-102723-065309 PMC12700611

[26] J. Xing , M. Tan , D. Leshchiner , M. Sun , M. Abdelgied , L. Huang , S. Paithankar , K. Uhl , R. Shankar , E. Lisabeth , B. Aleiwi , T. Jager , C. Lawson , R. Chen , M. Giletto , R. Girgis , R. R. Neubig , S. So , E. Ellsworth , X. Li , M.‐S. Chua , J. Zhou , B. Chen , Cell 2026, 189, 2556. 41850287 10.1016/j.cell.2026.02.016 PMC13041732

[27] A. G. Atanasov , S. B. Zotchev , V. M. Dirsch , I. E. Orhan , M. Banach , J. M. Rollinger , D. Barreca , W. Weckwerth , R. Bauer , E. A. Bayer , M. Majeed , A. Bishayee , V. Bochkov , G. K. Bonn , N. Braidy , F. Bucar , A. Cifuentes , G. D’Onofrio , M. Bodkin , M. Diederich , A. T. Dinkova‐Kostova , T. Efferth , K. El Bairi , N. Arkells , T.‐P. Fan , B. L. Fiebich , M. Freissmuth , M. I. Georgiev , S. Gibbons , K. M. Godfrey , C. W. Gruber , J. Heer , L. A. Huber , E. Ibanez , A. Kijjoa , A. K. Kiss , A. Lu , F. A. Macias , M. J. S. Miller , A. Mocan , R. Müller , F. Nicoletti , G. Perry , V. Pittalà , L. Rastrelli , M. Ristow , G. L. Russo , A. S. Silva , D. Schuster , H. Sheridan , K. Skalicka‐Woźniak , L. Skaltsounis , E. Sobarzo‐Sánchez , D. S. Bredt , H. Stuppner , A. Sureda , N. T. Tzvetkov , R. A. Vacca , B. B. Aggarwal , M. Battino , F. Giampieri , M. Wink , J.‐L. Wolfender , J. Xiao , A. W. K. Yeung , G. Lizard , M. A. Popp , M. Heinrich , I. Berindan‐Neagoe , M. Stadler , M. Daglia , R. Verpoorte , C. T. Supuran , the International Natural Product Sciences , Nat. Rev. Drug Discov. 2021, 20, 200. 33510482 10.1038/s41573-020-00114-z PMC7841765

[28] K. Ueda , C. J. H. Porter , A. Goodwin , L. S. Taylor , Nat. Rev. Drug Discov. 2026. 10.1038/s41573-026-01407-5 41927892

[29] H. Lu , Z. Ban , K. Xiao , M. Sun , Y. Liu , F. Chen , T. Shi , L. Chen , D. Shao , M. Zhang , W. Li , Adv. Sci. 2024, 11, 2308866. 10.1002/advs.202308866 PMC10933608 38196299

[30] Z. Wang , Y. Chen , H. Fang , K. Xiao , Z. Wu , X. Xie , J. Liu , F. Chen , Y. He , L. Wang , C. Yang , R. Pei , D. Shao , Sci. Adv. 2024, 10, eadp7022. 39485841 10.1126/sciadv.adp7022 PMC11529718

[31] F. Fang , X. Chen , ACS Nano 2024, 18, 23827. 39163559 10.1021/acsnano.4c09027

[32] L. Huang , S. Zhao , F. Fang , T. Xu , M. Lan , J. Zhang , Biomaterials 2021, 268, 120557. 33260095 10.1016/j.biomaterials.2020.120557

[33] C. Zhang , Y. Yuan , Q. Xia , J. Wang , K. Xu , Z. Gong , J. Lou , G. Li , L. Wang , L. Zhou , Z. Liu , K. Luo , X. Zhou , Adv. Sci. 2025, 12, 2415902. 10.1002/advs.202415902 PMC11884566 39792782

[34] H. Hu , C. Yang , M. Li , D. Shao , H.‐Q. Mao , K. W. Leong , Mater. Today 2021, 42, 99. 10.1016/j.mattod.2020.08.019 PMC8375602 34421329

[35] F. Cui , F. Chen , X. Xie , C. Guo , K. Xiao , Z. Wu , Y. Chen , J. Lu , F. Ruan , C. Cheng , C. Yang , D. Shao , Chin. Chem. Lett. 2024, 35, 108681.

[36] H. Hu , C. Yang , F. Zhang , M. Li , Z. Tu , L. Mu , J. Dawulieti , Y.‐H. Lao , Z. Xiao , H. Yan , W. Sun , D. Shao , K. W. Leong , Adv. Sci. 2021, 8, 2002020. 10.1002/advs.202002020 PMC8336609 34386315

[37] A. P. Davis , T. C. Wiegers , R. J. Johnson , D. Sciaky , J. Wiegers , Carolyn J. Mattingly , Nucleic Acids Res. 2023, 51, D1257. 36169237 10.1093/nar/gkac833 PMC9825590

[38] T. Liu , L. Hwang , Stephen K. Burley , Carmen I. Nitsche , C. Southan , W. P. Walters , Michael K. Gilson , Nucleic Acids Res. 2025, 53, D1633. 39574417 10.1093/nar/gkae1075 PMC11701568

[39] Z. Liu , M. Su , L. Han , J. Liu , Q. Yang , Y. Li , R. Wang , Acc. Chem. Res. 2017, 50, 302. 28182403 10.1021/acs.accounts.6b00491

[40] J. Gilmer , S. S. Schoenholz , P. F. Riley , O. Vinyals , G. E. Dahl , in Proc. 34th International Conference on Machine Learning 2017, 1263.

[41] Z. Lin , H. Akin , R. Rao , B. Hie , Z. Zhu , W. Lu , A. dos Santos Costa , M. Fazel‐Zarandi , T. Sercu , S. Candido , bioRxiv 2022, 2022, 500902.

[42] L. Chen , X. Tan , D. Wang , F. Zhong , X. Liu , T. Yang , X. Luo , K. Chen , H. Jiang , M. Zheng , Bioinformatics 2020, 36, 4406. 32428219 10.1093/bioinformatics/btaa524

[43] M. Tsubaki , K. Tomii , J. Sese , Bioinformatics 2019, 35, 309. 29982330 10.1093/bioinformatics/bty535

[44] Y. Igarashi , N. Nakatsu , T. Yamashita , A. Ono , Y. Ohno , T. Urushidani , H. Yamada , Nucleic Acids Res. 2015, 43, D921. 25313160 10.1093/nar/gku955 PMC4384023

[45] M. Kanehisa , M. Furumichi , M. Tanabe , Y. Sato , K. Morishima , Nucleic Acids Res. 2017, 45, D353. 27899662 10.1093/nar/gkw1092 PMC5210567

[46] D. Szklarczyk , R. Kirsch , M. Koutrouli , K. Nastou , F. Mehryary , R. Hachilif , A. L. Gable , T. Fang , Nadezhda T. Doncheva , S. Pyysalo , P. Bork , Lars J. Jensen , C. von Mering , Nucleic Acids Res. 2023, 51, D638. 36370105 10.1093/nar/gkac1000 PMC9825434

[47] M. J. Abraham , T. Murtola , R. Schulz , S. Páll , J. C. Smith , B. Hess , E. Lindahl , SoftwareX 2015, 1, 19.

[48] J. Zhang , T. Lu , Phys. Chem. Chem. Phys. 2021, 23, 20323. 34486612 10.1039/d1cp02805g

[49] F. Neese , F. Wennmohs , U. Becker , C. Riplinger , J. Chem. Phys. 2020, 152, 224108. 32534543 10.1063/5.0004608

[50] T. Lu , J. Chem. Phys. 2024, 161, 082503.39189657

[51] T. Lu , Sobtop 2025, 11.

[52] J. Wang , R. M. Wolf , J. W. Caldwell , P. A. Kollman , D. A. Case , J. Comput. Chem. 2004, 25, 1157. 15116359 10.1002/jcc.20035

