# Supplementary material for: AI‐enabled engineering of hesperidin/ursodeoxycholic acid nanomedicine for synergistic treatment of drug‐induced liver injury
Source: Smart Mol. 2026 Sep 3:e70101. Online ahead of print. doi: 10.1002/smo2.70101 (PMC13539393; doi:10.1002/smo2.70101)
Supplement: Supplementary file 1 [file SMO2-9999-0-s001.docx]

Supporting Information

AI-Enabled Engineering of Hesperidin/Ursodeoxycholic acid Nanomedicine for Synergistic Treatment of Drug-Induced Liver Injury

*Kai Xiao, Meng Fan, Haoyu Zheng, Boyuan Gu, Shaowen Wang, Ziping Wu, Shiming Zhang, Xin Zhao, Wensheng Zhang, Quanxin Ning, Sigen A, Chao Yang, Huayi Sun*, Ren Mo*, Dan Shao**

Supplementary Methods

*Reagents and materials:* Hesperidin (HES, ≥ 98%), ursodeoxycholic acid (UDCA, ≥ 98%) and dimethyl sulfoxide (DMSO) were purchased from Adamas Reagent Company (Shanghai, China). Dialysis bags (molecular weight cut-off: 3500 Da) were purchased from Viskase Companies (Houston, USA). Dulbecco’s modified Eagle’s medium (DMEM), fetal bovine serum (FBS), and penicillin-streptomycin were obtained from Gibco (USA). Acetaminophen (APAP) was purchased from Sigma-Aldrich (Germany). The Cell Counting Kit-8 (CCK-8) was purchased from APExBIO (Houston, USA). The LysoTracker Red were obtained from Beyotime (Jiangsu, China). Enzyme-linked immunosorbent assay (ELISA) kits for tumor necrosis factor-alpha (TNF-α) was obtained from BioLegend (San Diego, USA). DAPI was purchased from Beyotime (Shanghai, China). Malondialdehyde (MDA) content kits were obtained from Nanjing Jiancheng Bioengineering Institute (Nanjing, China). All other chemicals were of analytical grade and used without further purification.

*Target-Based API Screening*: The natural-product candidate library was obtained from TCMbank, and protein sequences of the selected target genes were retrieved from UniProt. More than 10,000 compounds were evaluated using the independently trained GSEC–GESM classification and affinity models. For each candidate API, all API-target pairs associated with the selected pathways were evaluated using the GSEC-GESM classification and affinity models. The predicted affinity values were integrated with the corresponding regulatory probabilities generated by the classification model to calculate pathway-level regulatory scores. For the cytokine-cytokine receptor interaction pathway, the predicted inhibitory probabilities were used as weights to prioritize compounds with stronger inhibitory potential. Similarly, activation probabilities were used to evaluate compounds targeting the glutathione metabolism pathway. The weighted scores from all pathway-associated targets were aggregated to rank candidate APIs, and the top-ranked compounds were selected for subsequent co-assembling test.

*Screening of Co-Assembling API Pairs*: The top-5 candidates from each therapeutic direction were evaluated in pairwise combinations. Each API was dissolved in DMSO at 20 mM. Under continuous stirring, the mixed organic solution was added dropwise to 20 volumes of deionized water to induce self-assembly. Hydrodynamic diameter and particle-size distribution were measured by dynamic light scattering using a Zetasizer Nano ZS.

*Preparation and Characterization of HUNMs*: HUNMs were prepared by flash nanocomplexation. HES and UDCA were separately dissolved in DMSO and introduced through two opposing mixer inlets at 2 mL min⁻¹ per inlet. Deionized water, at ten times the total DMSO volume, was introduced through the remaining two inlets at 20 mL min⁻¹ per inlet. The resulting nanosuspension was purified by dialysis against deionized water using a 3.5 kDa molecular-weight-cutoff (MWCO) membrane and subsequently freeze-dried. Particle morphology was examined by transmission electron microscopy at 200 kV. Hydrodynamic diameter, polydispersity index, and zeta potential were measured by dynamic light scattering. The relative contents of HES and UDCA were quantified by high-performance liquid chromatography (HPLC). HUNMs was dispersed in simulated small intestine fluid (Lea Gene), simulated gastric juice (Coolaber science & technology) and fetal bovine serum (Pricella Biotechnology) at a concentration of 0.1 mg/mL HUNMs to test *in vitro* stability. Size and PDI of HUNMs in different medium were measured at 0, 1, 2, 4 h.

*In Vitro Drug Release*: HUNMs (1 mg) were dispersed in phosphate-buffered saline at pH 7.4, transferred into dialysis bags with a MWCO of 3.5 kDa, and immersed in 1 L of release medium at 37 °C with shaking at 50 rpm. At predetermined intervals, aliquots were collected and replaced with an equal volume of fresh medium. Released HES and UDCA were quantified by HPLC.

*Detection of intracellular ROS*: HepG2 cells were seeded in 24-well plates at a density of 1 × 10^5^ cells per well. After cell attachment, cells were pretreated with HUNMs or free APIs for 4 h, followed by exposure to 400 µM H_2_O_2_ in serum-free DMEM for 6 h. Intracellular ROS levels were then detected by incubating the cells with DCFH-DA for 20 min at 37 °C. After washing three times with PBS, ROS levels were assessed using a fluorescence microscope.

**Figure S1.** Classification Matthews Correlation Coefficient (MCC) under warm-start (**a)**, protein cold-start (b) and API-scaffold cold-start conditions (c). area under precision-recall curve (AUPRC) under the corresponding warm-start (d), protein cold-start (e) and API-scaffold cold-start conditions (f).

**Figure S2.** Affinity-prediction performance of GSEC-GESM evaluated using root mean suqre error (RMSE) (a), mean square error (MSE) (b) and median absolute error (MedAE) (c) under the three cross-validation settings.

**Figure S3.** Gene-expression correlation matrix across time-matched control and APAP-treated liver samples collected at 3, 6, 9, and 24 h.

**Figure S4.** Volcano plots of differentially expressed genes in APAP-treated liver relative to the corresponding time-matched control at 3 h (a), 6 h (b), 9 h (c), and 24 h (d).

**Figure S5.** Predicted inhibitory scores of the top-5 intervention candidates across proteins encoded by the differentially expressed genes enriched in the cytokine–cytokine receptor interaction pathway.

**Figure S6.** Predicted activation scores of the top-5 intervention candidates across proteins encoded by the differentially expressed genes enriched in the glutathione metabolism pathway.

**Figure S7.** XRD patterns of HUNMs compared with physical mixture of HES and UDCA.

**Figure S8.** Time-dependent size (a) and PDI (b) evolution of HUNMs during incubation in PBS, simulated gastric fluid, simulated intestinal fluid, and fetal bovine serum over 4 h. Particle sizes were measured at indicated time points. Data are presented as mean ± SD (n = 3).

**Figure S9.** Representative snapshots of the molecular dynamics simulation at 20 ns, 200 ns, 300 ns, and 400 ns.

**Figure S10.** Time-dependent changes in the root-mean-square deviation (RMSD) of nonwater molecules (a) and the potential energy of the simulation system (b). Each panel contains three subplots corresponding to three independent parallel simulations.

**Figure S11.** One-dimensional density projection distributions of the final cluster components along the x- (a), y- (b), and z-axes (c). Each panel contains three subplots corresponding to three independent parallel simulations.

**Figure S12.** Molecular structure representations of HES and UDCA colored according to atomic electrostatic potential.

**Figure S13.** Molecular structure representations of HES and UDCA colored according to logP-based hydrophobicity distribution.

**Figure S14.** Semi-quantitative evaluation of HES cellular uptake by fluorescence imaging. The normalized fluorescence was calculated as the ratio of HES-FITC fluorescence area to DAPI-stained nuclear area. Data represent the mean ± SD (n=6). Statistical significance was assessed using a two-tailed unpaired t-test.
